# Polymorphisms in the proprotein convertase subtilisin/kexin type 9 gene in clinically diagnosed patients with familial hypercholesterolemia

**DOI:** 10.1590/1806-9282.20250873

**Published:** 2025-12-15

**Authors:** Aldrina Laura da Silva Costa, José Ernesto dos Santos, Wilson Salgado, Caroline Bertoncini-Silva, Letícia Bizari, Gustavo Santos Paiva Laender Moura, Raphael Del Roio Liberatore, Renato Augusto Zorzo, Vivian Marques Miguel Suen

**Affiliations:** 1 Universidade de São Paulo, Ribeirão Preto Medical School, Department of Internal Medicine – São Paulo (SP), Brazil.

**Keywords:** Familial hypercholesterolemia, Cardiovascular diseases, Gene polymorphisms, Proprotein convertase 9, Cholesterol, LDL

## Abstract

**OBJECTIVE:**

The aim of this study was to investigate polymorphisms in the proprotein convertase subtilisin/kexin type 9 gene and compare disease severity in familial hypercholesterolemia participants with and without these polymorphisms.

**METHODS:**

Fifty patients were included in this observational, cross-sectional study of primary data collection between June 2014 and June 2015 at Clinics Hospital of Ribeirão Preto Medical School/University of São Paulo Inclusion criteria for the study included individuals with an low-density lipoprotein cholesterol level ≥190 mg/dL, based on Dutch make early diagnosis prevent early death criteria for familial hypercholesterolemia. Patients with kidney disease, liver failure, a triglyceride level ≥300 mg/dL, or hypothyroidism were excluded. Gene analysis was performed using the high-resolution melting method. deoxyribonucleic acid samples with detected changes were sequenced to identify polymorphisms. Lipid profile, body mass index, history of cardiovascular events, gender, and statin use were compared between participants with and without polymorphisms.

**RESULTS:**

Of the 50 patients, 14 carried proprotein convertase subtilisin/kexin type 9 polymorphisms. Familial hypercholesterolemia was not associated with these polymorphisms in this cohort. As gene expression was not assessed, no conclusions can be drawn regarding functional effects. Six patients (four in the polymorphism group and two in the non-polymorphism group) reported cardiovascular events. The significantly higher occurrence of these events in the polymorphism group suggests a possible association between the presence of proprotein convertase subtilisin/kexin type 9 polymorphisms and an increased cardiovascular risk.

**CONCLUSION:**

Benign variants were identified in the proprotein convertase subtilisin/kexin type 9 gene, with no pathogenic alterations detected. Despite the significantly higher number of participants with a history of cardiovascular events in the polymorphism group, these data do not support a direct, causal association between these polymorphisms and an increased cardiovascular risk.

## INTRODUCTION

Cardiovascular diseases (CVDs) are the primary cause of death worldwide, contributing substantially to both mortality and disability. In 2021, they were linked to 20.5 million deaths (one-third of all deaths worldwide). Alarmingly, over three-quarters of these CVD-related deaths occurred in low- and middle-income countries^1^. Among the risk factors, hyperlipidemia stands out, especially familial hypercholesterolemia (FH) (OMIM 143890), one of the most common autosomal dominant genetic disorders. It is mainly characterized by high serum levels of low-density lipoprotein cholesterol (LDL-C) and early coronary artery disease (CAD)^2^.

The global prevalence of the heterozygous form (HeFH) of FH is estimated at 1:250 to 1:300 cases; in contrast, the much rarer homozygous form (HoFH) occurs at a frequency of 1:250,000 to 1:360,000 cases^3,4^. Recent data from 2023 estimates that FH affects approximately 1% of Brazilian adults—more precisely, one in every 104 adults may have a possible diagnosis of FH^5^.

FH is predominantly caused by the presence of loss-of-function variants in the LDL receptor (LDLR) gene (OMIM: 606945). Less common causes are loss-of-function variants in the apolipoprotein B (APOB) gene (OMIM: 107730) and gain-of-function (GOF) variants in the proprotein convertase subtilisin/kexin type 9 (PCSK9) gene (OMIM: 607786) or apolipoprotein E (APOE) gene (OMIM: 107741). In rarer autosomal forms, pathogenic variants in the low-density lipoprotein receptor adaptor protein 1 (LDLRAP1) gene (OMIM: 605747) may also be involved^6^.

Over 2000 mutations of the gene have been identified in the LDLR gene, including deletions, insertions, missense, copy number variants, and nonsense mutations^7^.

Unlike the LDLR gene, which exhibits extensive mutational heterogeneity, the number of confirmed pathogenic variants in the APOB gene is significantly lower, with the p.Arg3527Gln variant being the most common^8^. Although few GOF mutations have been identified in the PCSK9 gene, more than 850 mutations have already been reported. Notably, among these are the loss-of-function variants, which confer a protective effect^9^. Mutations in autosomal recessive genes, such as LDLRAP1 and APOE, are very rare and are typically found in more severe forms or in specific populations^10^.

In 2003, the PCSK9 gene was identified as the third gene implicated in autosomal dominant hypercholesterolemia^11^. It is located on chromosome 1p32.3 and comprises 15 exons. It encodes a precursor protein of 692 amino acids, composed of four key regions: an N-terminal signal peptide (residues 1–30), a prodomain (31–152), a catalytic domain (153–425), and a C-terminal cysteine-histidine-rich (CHR) domain (426–692). After autocatalytic processing in the endoplasmic reticulum, the prodomain remains non-covalently bound, inhibiting protease activity and enabling the mature PCSK9 to function as a chaperone that directs the LDLR to lysosomal degradation rather than recycling. This reduces LDLR expression on hepatocyte surfaces, impairing LDL-C clearance and leading to elevated plasma LDL-C levels^12^.

The well-characterized D374Y mutation, located in the catalytic domain, enhances PCSK9's binding affinity for the epidermal growth factor-like repeat A domain of LDLR by 10- to 25-fold, causing severe hypercholesterolemia and markedly increased risk of early CAD^13,14^. Other GOF mutations—such as S127R in the prodomain and variants in the CHR domain like R496W—disrupt PCSK9's binding to LDL particles, which normally serve to inhibit its activity. This disruption exacerbates LDLR degradation and LDL-C elevation, culminating in a higher risk of CAD^10,11,15^. This discovery paved the way for the development of PCSK9 inhibitors, representing a major advancement in the treatment of FH.

In Brazil, despite the burden caused by CVDs, data on the genetic background of FH are limited. Borges et al.^16^ identified 27 deleterious variants in LDLR in the Brazilian FH cohort FHBGEP (Genomics, Epigenomics and Pharmacogenomics of Familial Hypercholesterolemia [Brazil Project]). Studies that investigated polymorphisms in the PCSK9 gene in FH in Brazil are still limited^17^. Therefore, this study aimed to detect polymorphisms in the PCSK9 gene and compare disease severity among participants clinically diagnosed with FH, who either carried polymorphisms or not, at an outpatient clinic of a public tertiary hospital in Southeastern Brazil.

## METHODS

### Subject recruitment

This was a cross-sectional observational study with convenience sampling. The STROBE (Strengthening the Reporting of Observational Studies in Epidemiology) checklist was followed. A 1-year screening of 17,017 lipid profiles identified 480 individuals with LDL-C ≥190 mg/dL, based on Dutch Lipid Clinic Network (Dutch MEDPED) criteria^18^ for FH. After excluding patients with kidney disease, liver failure, triglycerides ≥300 mg/dL, or hypothyroidism, 218 remained with possible FH and were contacted by telephone. Among them, 40 participants agreed to participate. In addition, the 10 first-degree relatives of the initially recruited who met the same diagnostic criteria were included, totaling 50 participants eligible for the study.

The study was approved by the research committee of the Clinical Hospital of Ribeirao Preto Medical School of USP—CAAE: 46492313.3.0000.5440 and performed in accordance with the Declaration of Helsinki. It also complied with the Standards and Guidelines for Research Involving Human Subjects in Brazil (CNS Resolution 466/2012 and CNS Resolution 510/2016). All participants provided written informed consent.

### Proprotein convertase subtilisin/kexin type 9 gene mutation analysis

#### Biochemical analysis, anthropometric, and nutritional evaluation

After a 12-h fast, 10 mL of blood was collected in an ethylenediaminetetraacetic acid tube for plasma lipid analysis and deoxyribonucleic acid (DNA) extraction. Next, bioelectrical impedance analysis was performed (Biodynamics^®^, model 450), providing total body resistance and reactance. Weight was measured with a Filizola^®^ electronic scale (capacity 150 kg, precision 0.1 kg), height with a wall-mounted stadiometer (precision 0.5 cm), and waist circumference with a non-elastic tape, according to standardized methods for adults^19^ and youth^20^. Participants also completed questionnaires on health habits and food frequency.

#### Deoxyribonucleic acid extraction

Genomic DNA was extracted from 2.5 mL of whole blood using the Maxwell Mdx system with the Promega kit (AS1010), following the manufacturer's protocol. DNA samples were stored at −80°C in the Molecular Genetics and Bioinformatics Laboratory, Department of Genetics, Ribeirão Preto Medical School, University of São Paulo.

#### Polymerase chain reaction

DNA samples, adjusted to a concentration of 100 ng/μL, were initially analyzed by PCR to optimize primer annealing temperatures prior to subsequent high-resolution melting (HRM) analysis.

#### High-resolution melting

HRM analysis was performed using conventional PCR reagents supplemented with the fluorescent DNA intercalating dye MeltDoctor (Applied Biosystems). This fluorophore exhibits maximum fluorescence intensity (100%) when DNA is in its double-stranded state. Mutations were detected by fluorescence decrease with rising DNA melting temperature, producing genotype-specific melting curves. The optimal detection point was defined at 50% fluorescence decay.

Reactions were prepared in a total volume of 20 μL, containing: 3.6 μL of Milli-Q water, 1.2 μL of each 5 μM primer, 10 μL of MeltDoctor HRM^TM^ Master Mix (Applied Biosystems), and 4 μL of DNA at 4 ng/μL. All reactions were run in duplicate.

Thermocycling parameters for the amplification step were: 95°C for 10 s, followed by 40 cycles of 95°C for 15 s and annealing at the appropriate temperature for 1 min. For melting curve analysis, the parameters were: 95°C for 10 s, 60°C for 1 min, 95°C for 15 s, and 60°C for 15 s. Results were analyzed using the Applied Biosystems^®^ 7500 Fast Real-Time PCR System and Applied Biosystems^®^ HRM Software v2.0. Samples displaying similar melting curves were grouped as variants. Each variant group was then compared with wild-type samples based on the following criteria: (i) differences in melting curve patterns between duplicates, (ii) shifts in melting temperature, and (iii) alterations in overall melting curve profile^21^.

#### DNA sequencing

The primer sequences were designed based on the gene sequence available in the GenBank database (access number: NM_174936), and the maximum programmed fragment size was 393 bp.

Amplifications were performed with a total volume of 25 μL containing 14.5 μL of milliQ water, 2.5 μL of 10× buffer, 2 μL of 1.24 mM deoxyribonucleoside triphosphate, 1.5 μL for each primer (forward and reverse) at 2.5 pmol/μL, 1.0 μL of 1 U Taq DNA polymerase (Biotools, Madrid, Spain), and 2 μL of DNA.

The products of amplification by PCR were submitted to automated capillary sequencing using the 3500 XL Genetic Analyzer (Applied Biosystems). The sequences were analyzed using Sequencing Analysis software, version 5.2. The primer sequences were described in Table 1.

**Table 1:** Primers for polymerase chain reaction amplification of proprotein convertase subtilisin/kexin type 9 exons.

Amplified region	Sequence	Size (bp)
**Exon 1**	FP- 5′-TGGACCGTGCACGGCCTCTAGGT-3′ RP- 5′-ACCGTCCTGGCTGCTGGTTCGC-3′	315
**Exon 2**	FP- 5′-GGGTGAGATAAAGTACACCTAGG-3′ RP- 5′-CCCTATCAGGAAGTGCCATTCC-3′	304
**Exon 3**	FP- 5′-GTCCAAATGGCTTAAGCAGAGTCC-3′ RP- 5′-GCAGAGCAAATGGATTCAGCTCAG-3′	229
**Exon 4**	FP- 5′-GGTTGACTTTATGCTCATTCCCTC-3′ RP- 5′-TACAGCTGCAACGCTCTGTGGTG-3′	278
**Exon 5**	FP- 5′-TTCATCCATCCAGCCACCTGCTGA-3′ RP- 5′-AGGTCCAGATGGAGAGAGACCAG-3′	266
**Exon 6**	FP- 5′-AACCATCACTCTGTGCCTGTAAGG-3′ RP- 5′-ACAAGAAGCCCACGTGCCACAAGA-3′	389
**Exon 7**	FP- 5′-CTCTCTTGGGCTCCTTTCTCTGC-3′ RP- 5′-TGTTAGCATCACGGTGGTCGAGG-3′	272
**Exon 8**	FP- 5′-GCCATCACCATCTTTCACCATTCA-3′ RP- 5′-AAGGGAGAGACTGTCAAGGTCACA-3′	285
**Exon 9**	FP- 5′-ATTTCTGTGGAGGTCCCCTCACTC-3′ RP- 5′-ACACGAAGGAGGGTTACAGTCACC-3′	183
**Exon 10**	FP- 5′-CAAAGCCCATTCTAAAGCAGATTCC-3′ RP- 5′-AGTGGGTGCATAAGGAGAAAGAGAC-3′	284
**Exon 11**	FP- 5′-GAGACGGAGCATCCCAGCATTTCA-3′ RP- 5′-CAGAAAAGCTGTGCAGGAGAGACA-3′	297
**Exon 12**	FP- 5′-TTTTCCTCGGGCTCTGGCAGGTGAC-3′ RP- 5′-TTGACCCTCCCCAGACACCCATCCT-3′	273

FP: forward primer; RP: reverse primer.

#### Classification of variants

After initial detection by HRM and sequencing, variant analysis and classification were performed using genetic databases, including Database of Single Nucleotide Polymorphisms, ClinVar, and gnomAD, in addition to the American College of Medical Genetics and Genomics recommendations for defining pathogenicity. Chromatogram validation and interpretation were performed using FinchTV software (Geospiza, Inc.).

### Statistical analysis

Continuous variables were expressed as mean±standard deviation and categorical variables as percentages. Group comparisons were performed with independent samples t-tests. Statistical significance was defined as p<0.05 (Statistical Package for the Social Sciences [SPSS] V22).

## RESULTS

DNA sequencing identified seven distinct variants across the 12 exons of the PCSK9 gene in the 50 participants, totaling 600 exon analyses. Fourteen individuals carried these variants, accounting for 17 polymorphisms overall (Table 2). Exon 12 was the only exon to exhibit two distinct variants. All variants were classified as benign polymorphisms. Among the carriers, one participant presented three distinct alterations and another presented two. In total, the 17 polymorphisms corresponded to seven previously unreported (novel) variants. Notably, participant 10 exhibited three polymorphism-type alterations and had an LDL cholesterol level above 250 mg/dL despite ongoing lipid-lowering therapy.

**Table 2:** Variants identified# in the proprotein convertase subtilisin/kexin type 9 gene.

Description	Exon	Base transition	Nucleotide triplet	Amino acid change	Number of polymorphisms found	Type of genetic variant	Participants*
p.Glu57Lys	1	G/A	GAG>AAG	Glu → Lys	1	Polymorphism	13
p.Gly240GIy	5	C/T	GGC>GGT	Gly → Gly	1	Polymorphism	10
p.Gln342Gln	7	A/G	CAA>CAG	Gln → Gln	8	Polymorphism	1,2,5,7,8,9,11,12
p.Val474IIe	9	G/A	GTC>ATC	Val → IIe	4	Polymorphism	3,4,10,14
p.Ala617Ala	11	A/C	CAC>CCC	Ala → Ala	1	Polymorphism	6
p.His644His	12	C/T	CAC>CAT	His → His	1	Polymorphism	10
p.Gly670Glu	12	A/A	GGG>GAG	Gly → Glu	1	Polymorphism	13
Total	7	-	-	-	17	-	14

# The seven variants identified in the 14 participants were novel, as they had not been previously reported.

* Participants are numbered one through 14, representing the 14 participants who carried polymorphisms. GAG: Guanine–Adenine–Guanine; GGC: Guanine–Guanine–Cytosine; CAA: Cytosine–Adenine–Adenine; GTC: Guanine–Thymine–Cytosine; CAC: Cytosine–Adenine–Cytosine; GGG: Guanine–Guanine–Guanine; AAG: Adenine–Adenine–Guanine; GGT: Guanine–Guanine–Thymine; CAG: Cytosine–Adenine–Guanine; ATC: Adenine–Thymine–Cytosine; CCC: Cytosine–Cytosine–Cytosine; CAT: Cytosine–Adenine–Thymine.

There were no significant differences in most of the variables between the group with polymorphisms and the group without polymorphisms, except for total cholesterol (Table 3).

**Table 3:** Comparison of age, body mass index, body fat percentage, gender, and age between participants with and without polymorphisms in the proprotein convertase subtilisin/kexin type 9 gene.

Participants characteristics	Polymorphism n=14 (28%)	No polymorphism n=36 (72%)	p-value
Age (years)	35.1±15.8	39.0±22.1	0.316
Men	11 (78.6%)	15 (41.7%)	–
Women	3 (21.4%)	21 (58.3%)	–
BMI (kg/m^2^)	29.8±9.2	25.7±5.1	0.308
% of BF	27.0±9.2	28.7±5.8	0.645
Total cholesterol (mg/dL)	212.8±31.9	259.2±72	0.031
HDL-c (mg/dL)	41.6±10	41.0±8.6	0.154
LDL-c (mg/dL)	192.1±71.1	151.1±36.4	0.146
Use of lipid-lowering drugs	10 (71.4%)	22 (61.1%)	–
Cardiovascular event	4	2	0.02

Results presented as means (standard deviation) or count (percentage), as appropriate. BMI: body mass index; BF: body fat; HDL-c: high-density lipoprotein cholesterol; LDL-c: low-density lipoprotein cholesterol. BF percentage was obtained from bioelectrical impedance analysis (BIA). p-value—significance level obtained through Student's t-test analysis.

Among the participants with a history of HF, six reported one or more early CVEs (verified from electronic medical records). In the group with polymorphisms, four men reported events such as cerebrovascular accidents, acute myocardial infarction, or thrombosis before the age of 55. In the group without polymorphisms, two women also reported a history of CVEs before the age of 60.

## DISCUSSION

This study highlights the relevance of advancing genomic research within the country. Although previous literature has extensively examined mutations and polymorphisms in the PCSK9 gene, our investigation fills a critical gap by being, to our knowledge, among the first to compare disease severity among individuals diagnosed with FH. A distinctive strength of our work is its focus on a population managed in a tertiary-care hospital, the setting where this condition is most often identified. This approach provides a more comprehensive understanding of the genetic landscape of FH in a real-world clinical context, offering valuable insights to guide future diagnostic and therapeutic strategies.

Los et al.^22^ investigated PCSK9 gene variants in Brazilians with FH and identified nine variants in the coding region, including E32K and R469W, which were selected for functional analyses. The E32K variant showed a possible association with FH in a Brazilian family of Japanese descent. Functional assays revealed that the E32K and R469W variants reduced LDLR activity by 5 and 11%, respectively, compared to wild-type PCSK9; however, these reductions in LDLR activity and LDL internalization in transfected cells were not statistically significant. The 2021 update of the Brazilian guideline on FH^3^ emphasizes that GOF mutations in the PCSK9 gene, though less common, are associated with elevated LDL-C levels and increased cardiovascular risk. Taken together, these findings suggest that, although rare, such mutations play a significant role in the pathophysiology of FH, particularly in genetically diverse populations such as the Brazilian population.

Despite the presence of mutations in any of the three common genes related to FH, they do not account for all cases^3,4^. PCSK9 is a highly polymorphic gene, and its polymorphisms and mutations have been linked to variability in LDL-C levels and CAD. A common single-nucleotide polymorphism, rs505151 (E670G), located in the cysteine-rich C-terminal domain of exon 12, results in a substitution of glutamate for glycine and is potentially associated with a change in the enzymatic activity of PCSK9^23^. Lin et al.^23^ found that the PCSK9 E670G polymorphism was significantly correlated with the development of CAD in 225 hospitalized individuals, particularly in individuals with the G allele and in the dominant allele.

Considering that pathogenic mutations in the PCSK9 gene have been frequently observed in individuals of Portuguese descent—a relevant finding given the genetic contribution of this population to the Brazilian genomic background—it is noteworthy that no such variant was identified in the present study. These individuals, called FH phenocopies, have an increased risk of atherosclerotic CVDs compared to individuals with normal lipid levels. However, it is important to note that this risk is lower than that observed in individuals with monogenic forms of the disease^3^.

In the present study, analysis of the polymorphisms in the PCSK9 gene revealed that, unlike pathogenic variants for FH, these are generally considered benign or functionally neutral in the literature. For example, synonymous variants do not alter the protein, while the missense mutations, like p.Val474Ile and p.Glu57Lys, are common in the general population and show no functional impact^10^. While the p.Gly670Glu variant presents conflicting data, its functional impact is significantly lower than that of mutations that cause FH^10^. The absence of individual pathogenicity and the high population frequency of these polymorphisms suggest they are not the primary cause of FH^24^. However, their reported association with an increased cardiovascular risk indicates that they may act as cardiovascular risk modifiers, contributing to a polygenic risk profile rather than a monogenic cause for the disease.

Interestingly, the average age at the onset of coronary symptoms is lower in men (55 years) than in women (60 years). This is consistent with our findings, as the four men and two women experienced CVEs below these average ages, respectively. Furthermore, among the participants with a history of CVE, four were in the group with polymorphisms, a difference that was statistically significant, indicating a strong association between these genetic variations and early CVE.

Curiously, in the present study, although the number of participants with polymorphisms was smaller, there was a higher incidence of CVE. This finding suggests that these participants might have mutations in the LDLR or ApoB genes. The number of mutations described in the PCSK9 gene is relatively low, representing less than 1% of the mutations in the cases. However, with the development of new drugs that target the PCSK9 gene by reducing its function, it is important to describe the genetic profile of this population in order to find pathogenic mutations related to this gene. Identifying pathogenic mutations related to this gene can pave the way for safer and more effective treatments for affected individuals.

This study has limitations. First, the small sample size, which resulted from logistical constraints related to the availability of participants who consented to attend the hospital during the scheduled study period. Second, as this was an observational cross-sectional study, causal relationships cannot be established from the observed associations. Additionally, the possibility of selection and information biases cannot be excluded. However, it is important to highlight that this study is among the first to investigate polymorphisms in the PCSK9 gene, providing a foundation for future research on FH.

## CONCLUSION

This study identified a benign gene variant in the PCSK9 gene without detecting any pathogenic alterations. Although the number of participants with a history of CVE was significantly higher in the group with polymorphism, there is no evidence supporting a direct association between these polymorphisms and an increased risk of such events.

## Data Availability

The datasets generated and/or analyzed during the current study are available from the corresponding author upon reasonable request.
